# Comparison of immunohistochemistry (IHC) and fluorescence in situ hybridization (FISH) assessment for Her-2 status in breast cancer

**DOI:** 10.1186/1477-7819-7-83

**Published:** 2009-11-09

**Authors:** Weiguo Sui, Minglin Ou, Jiejing Chen, Youhua Wan, Hongbo Peng, Minfang Qi, He Huang, Yong Dai

**Affiliations:** 1 Laboratory Center of Guangzhou Military Area Command, 181st Hospital of People's Liberation Army, Guilin, Guangxi, PR China; 2 College of Life Science, Guangxi Normal University, Guilin, Guangxi, PR China; 3 Pathology Department of Guangzhou Military Area Command, 181st Hospital of People's Liberation Army, Guilin, Guangxi, PR China

## Abstract

**Background:**

The concordance rate between IHC and FISH according to clinical performance is still controversial. We report a prospective study to reflect the concordance between IHC and FISH in Guilin city, People's Republic of China.

**Methods:**

Fifty cases of invasive ductal carcinoma of breast tested by IHC and scored as 0, 1+, 2+ and 3+ by pathologists were further analyzed by FISH using a commercially available double-color probe, and the FISH findings were compared with IHC test results.

**Results:**

A total concordance of 82.0% was observed with a Kappa coefficient of 0.640 (P < 0.001). A high discordance was observed in 30.0% of the patients with IHC 2+, 7.1% in IHC 3+, 19.2% overall in IHC 0 and 1+.

**Conclusion:**

The IHC can be used firstly to screen the HER-2 status, and FISH can be used as a supplementary role to IHC and 2+ and some negative cases. And only those cases with Her-2 status of IHC 3+ or FISH positive should be treated with Herceptin.

## Background

Breast cancer is one of the most common malignancy in the world. According to the global cancer statistics, Europe and America has the high incidence and mortality of breast cancer [1]. The incidence of breast cancer in China is 20 per 100,000 population, and the incidence is growing [2]. Researches have shown that about 20%-30% of the breast cancer patients have Her-2 amplification or over expression, that is associating with a more aggressive phenotype and decreased survival [3-7]. The benefit of humanized anti-Her-2 monoclonal antibody trastuzumab (Herceptin) in Her-2-positive breast cancers has been well documented as noted by prolonged survival [8]. But this therapy is effective only if the detection of Her-2 status is accurate.

There are several methods available to detect the Her-2 status like polymerase chain reaction (PCR), immunohistochemistry (IHC), fluorescence *in situ *hybridization (FISH), chromogenic in situ hybridisation (CISH) [9,10]. Protein over-expression detected by IHC or amplification of Her-2 gene analyzed by FISH are the two main methods used to detect Her-2 status in clinical practice. FISH is considered as a gold standard because of its sensitivity and specificity. But FISH has disadvantages as it requires a modern and expensive fluorescence microscope equipped with multi-band-pass fluorescence filters, and the fluorescence fades so quickly that it could not provide a permanent record [10]. Compared with FISH, IHC is widely used in china as it is cheaper and convenient to operate and conserve; the morphology is clear. Comparative studies of IHC and FISH have generally shown a high concordance rate by some researches [11]. But protein overexpression may be found without gene amplification or gene amplification can be found in negative IHC [12]. Research has documented that the discordance rate between Her-2 by FISH and IHC is high in all four IHC scores (0, 1+, 2+, 3+), and a FISH-alone screening strategy has been alternatively suggested [13]. Our objective was to perform a prospective study in our own local setting and record the concordance between IHC and FISH in 50 cases of invasive ductal carcinoma of breast.

## Methods

### Study Design

The study population consists of 50 cases of invasive ductal carcinoma of breast treated between July, 2008 and March, 2009 at The 181 Hospital and Traditional Chinese Medicine Hospital which are the two main hospitals for the treatment of breast cancer in Guilin city of China. The specimens were fixed in 10% neutral-buffered formalin (pH7.4) for 24 hours. Only cases with sufficient invasive carcinoma for multiple assays were included in the study. For each case, 2-4 μm thick tissue sections were cut from a representative paraffin block and applied to positively charged slides. Her-2 protein expression was measured using a commercial available S-P kit. FISH for Her-2 gene amplification was performed in the key laboratory of 181 Hospital using a commercial available double-color probe. The interpretation of IHC and FISH were each performed by investigators blinded to the results of the other assay

### IHC analysis

IHC study was performed on paraffinem-bedded, formalin-fixed tissue sections using a commercial available Ultra Sensitive™ S-P kit (Maixin-Bio Co., Fuzhou, China), following the manufacturer's instructions and American Society of Clinical Oncology/College of American Pathologists guideline recommendations for human Epidermal Growth Factor Receptor 2 testing in breast cancer [14]. Briefly, this procedure included the deparaffinization and rehydration steps, followed by an epitope retrieval step in which the tissue sample was incubated in a citrate buffer solution at 90-95°C for 20 minutes. The slides were then subjected to a series of alternating washes in tris (hydroxymethyl) aminomethane hydrochloride buffer and incubation steps with, first, a peroxidase-blocking reagent for 5 minutes and then with Her-2 primary antibody, followed by a visualization reagent for 30 minutes each, and finally with a 3,3'-diaminobenzidine chromogen solution. After a finally wash, the slides were counterstained with haematoxylin [15].

**Figure 1:**
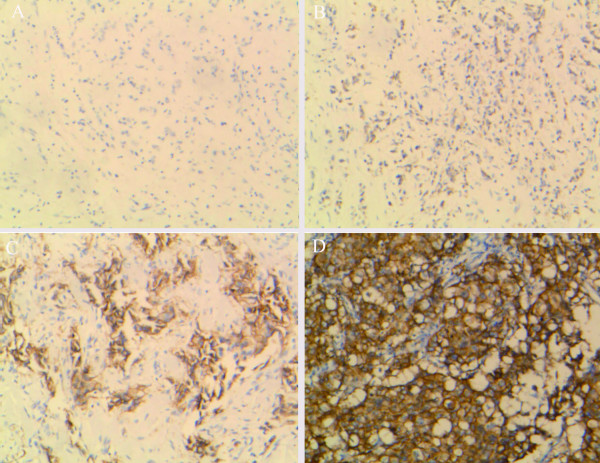
**IHC for Her-2 protein expression**. Figure 1 (A) Completely negative, IHC0; (B) Faint membranous positivity, ICH1+; (C) Moderate membranous positivity, IHC2+; (D)Strong, more than 30% tumor cells with circumferential membranous positivity, IHC3+.

Tumor cells with circumferential membranous positivity were considered as Her-2 protein over expression, and scoring was performed according to the manufacturer's recommendations by pathologists in a number of different practice groups (Figure 1), each with at least 10 years of experience in clinical practice; in order to truly reflect the concordance or discordances between IHC and FISH in our daily practices, the results of IHC were all from the data bank of the two hospitals and without revision again.

### FISH for Her-2 gene amplification

FISH analysis was modified in cooperation with the manufacturer of China Medical Technologies, Inc. (Beijing, China). The commercially available double-color FISH probe consisted of two probes: 17q11.2-q12 (labeled with Spectrum Orange) covering the whole Her-2 gene and the control, centromeric chromosome 17p11.1-q11.1 (labeled with Spectrum Green). The FISH fixed glass microscope slides with tissue sections were baked overnight at 65°C, deparaffinized in two 10-minute changes of xylene, transferred through two 3-minute changes of 100% ethanol, one 3-minute changes of 85% ethanol, one 3-minute changes of 70% ethanol and immersed for 15 minutes in pure water at 90°C. The slides were then incubated for 7-15 minutes in protease solution at 37°C. Then the slides were briefly washed in 2× sodium saline citrate (2× SSC; pH 7.2) at room temperature, dehydrated through 70%, 85%, 100% ethanol and acetone, then allowed to air dry. To denature DNA, the slides were placed in 78.5°C preheated 70% formamide/2× SSC for 8 min and then dehydration in a graded series of concentrations of ethanol which were precooling in -20°C. After drying in the open-air, 10 μl of probe which was destructured at 75.5°C for 7 min was applied onto each slide, cover slip was placed and sealed with rubber cement, then hybridized overnight at 42.8°C. After 16-18 h of hybridization, the slides were washed in 46°C preheated post-hybridization buffer (2× SSC/0.1% sodium dodecyl sulfate) for 5 min and rinsed in 70% ethanol. After air-drying (out of direct light), the slides were counterstained with 15 μL DAPI/anti-fade solution and cover slip applied.

**Figure 2:**
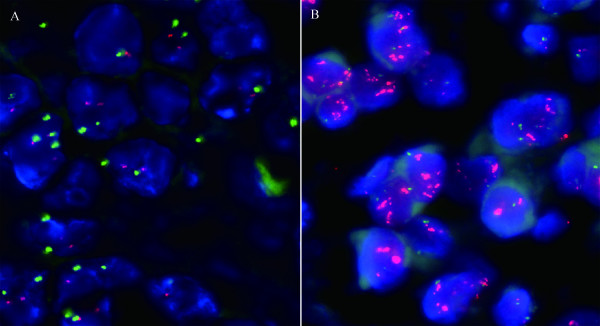
**FISH for Her-2 gene amplification**. Figure 2 (A) Negative amplification of human epidermal growth factor receptor-2/neu (Her-2) gene case with the ratio of Her-2 (red signals) to CEP 17 (green signals) is obviously smaller than 1.8; (B) Positive amplification of human epidermal growth factor receptor-2/neu (Her-2) gene case with the ratio of Her-2 (red signals) to CEP 17 (green signals) is obviously larger than 2.2.

FISH analysis was performed by two cytotechnologistes who were blinded to the clinical diagnoses at the time of evaluation. The slides were scanned using a OLYMPUS BX51 fluorescent microscope (OLYMPUS BX51, Japan) equipped with a 100-watt mercury lamp and single band pass filter set to detect DAPI, Rhodamine (17q11.2-q12), and FITC (chromosome 17) at 1000×. Thirty randomly selected invasive tumor nuclei in each of two separate, distinct microscopic areas were evaluated. Cases were scored as negative by FISH when the Her-2 with a Her-2 to CEP 17 ratio < 1.8 by counting at least 30 interphase nuclei, and those cases with a Her-2 to CEP 17 ratio > 2.2 were scored as positive (Figure 2). In addition, more randomly selected invasive tumor nuclei (for example, a total of 100 nuclei) would be evaluated if the Her-2 with a Her-2 to CEP 17 ratio was between 1.8 and 2.2.

## Results

Of the 50 specimens in our study (invasive ductal carcinoma with varying tumor grades and clinical stages), 9 were classified as IHC 0, 17 were classified as IHC 1+, 10 were classified as IHC 2+, and 14 were classified as IHC 3+. Five of the IHC 0 and 1+ cases, seven of the 10 IHC 2+ cases and 13 of the 14 IHC 3+ cases were found to be Her-2 FISH positive. They had a total concordance of 82.0% and a Kappa coefficient of 0.640 (P < 0.001), which was defined as IHC 2+/3+ and Her-2 FISH positive, or IHC 0/1+ and Her-2 FISH negative.

Discordance was defined as a discrepancy between the IHC and Her-2 FISH, including the following two conditions: (1) IHC 2+ or 3+ but Her-2 FISH negative; (2) IHC 0 or 1+ but Her-2 FISH positive. For example, the discordance rate according to IHC 0 and 1+ was defined as the number of discrepant IHC 0 and 1+ cases divided by the total number of IHC 0 and 1+ cases and was 19.2% (5/26). Following the same way of counting, the discordance rate according to IHC 2+ was 30.0% (3/10), IHC 3+ was 7.1% (1/14). The overall discordance rate by IHC was therefore 18.0% (9/50) (Table 1).

**Table 1:** Comparison of the results of IHC and FISH

**IHC scoring**	**Her-2 FISH amplified**	**Her-2 FISH non-amplified**	**Concordance by IHC**	**Discordance by IHC**
0 and 1+ (n = 26)	5	21	(21/26)80.8%	(5/26)19.2%
2+ (n = 10)	7	3	(7/10)70.0%	(3/10)30.0%
3+ (n = 14)	13	1	(13/14)92.9%	(1/14)7.1%

IHC = immunohistochemistry; FISH = fluorescence in situ hybridization

## Discussion

Reliable laboratory data in evaluating Her-2 status is essential, because the treatment is beneficial for advanced breast cancer and can avoid potential cardiotoxic effects in women not showing amplification or overexpression [16]. Her-2 status studied at the levels of DNA using FISH and protein using IHC are the two most accessible and feasible methods used in clinical diagnosis, and certain kits or antibodies are approved by the FDA (U.S. Food and Drug Administration). IHC is easy to perform and relatively cheap, and is predominantly used to evaluate Her-2 status. However, a wide range of sensitivity and specificity has been observed among various commercially available antibodies [17]. As an alternative, FISH is also recognized as a modality in cases with an equivocal IHC status with higher sensitivity and specificity.

Considering FISH as a gold standard, research reports that positive FISH results could be found in 91.7%, 23.2%, 7.4% and 4.1% in cases with IHC respectively diagnosed as 3+, 2+, 1+ and 0 [18]. In addition, some clinical trials have shown that amplification by FISH is more predictive of response to trastuzumab than IHC [12,19,20].

Our objective in this study was not to try and compare the sensitivity and specificity of these two tests (IHC and FISH). This study aimed to investigate the concordance and discordance rates between IHC and Her-2 FISH. Some similar studies adopting a similar strategy have been reported. Dolan and Snover found that the concordance between the IHC and FISH scores (defined as cases that were IHC negative/FISH nonamplified or IHC positive/FISH amplified) was found in 35 cases (27.1%) and discordance in 94 cases (72.9%) [15]. Lan *et al*. used FISH to ascertain the prevalence of erb-b2 gene amplification in 221 cases of breast cancer specimens read as 2+ in IHC analysis, and found 96 (44.4%) cases were detected to be erb-b2 amplified [21]. Kuo *et al*. compared FISH and IHC in breast cancer patients and found that the discordance rates by IHC were high (46.7% in IHC 2+, 16.7% in IHC 3+, 30.3% overall in IHC 2+ or 3+) [22]. All these researches indicated that the concordance between IHC and FISH is still controversial.

There are some factors leading to false IHC test results, including variability in tissue fixation and processing, variable sensitivity and specificity of commercially available antibodies, and differences in scoring criteria with considerable interobserver variability in interpretation of results [23]. Our study is trying to answer a simpler question about the concordance between IHC and FISH in our local setting in Guilin city of China, using some commercially available antibodies, which have not been approved by the FDA. Compared with some studies [15,22,23], our concordance in the cases of IHC 3+ is similar with theirs, but there are some differences in the cases of IHC 0, 1+, 2+. The concordance in this study of IHC 0 and 1+ is lower, and IHC 2+ is higher than theirs. This may due to the different sensitivity and specificity of the antibodies and probe used in this study. The different sensitivity and specificity of the antibodies and probe need to be further researched.

## Conclusion

In order to improve the therapeutic effect of herceptin in Her-2-positive breast cancers, the current algorithm using FISH as a supplementary role to IHC 2+ need to be modified according to this study in our setting. The IHC can be used firstly to screen the HER-2 status, and FISH can be used as a supplementary role to detect IHC and 2+ and some negative cases, especially those with a high tumour grades. And only those cases with Her-2 status of IHC 3+ or FISH positive are proposed to be treated with herceptin.

## Competing interests

This work is supported by the funding of Ministry of Health, P. R. China (Funding NO. WKJ 2007-3-001).

## Authors' contributions

WS carried out the studies design, helped to draft the manuscript. MO drafted the manuscript and participated in FISH analysis. JC and HP carried out FISH analysis. YW, MQ and HH carried out IHC analysis. YD conceived of the study, and participated in its design and coordination and helped to draft the manuscript. All authors read and approved the final manuscript.
